# Exploring resilience mechanism in learning burnout among pupils: school adjustment and academic self-efficacy

**DOI:** 10.3389/fpsyg.2025.1706567

**Published:** 2025-11-14

**Authors:** Changcheng Jiang, Qiufeng Gao

**Affiliations:** School of Government, Shenzhen University, Shenzhen, China

**Keywords:** learning burnout, resilience, school adjustment, academic self-efficacy, pupils

## Abstract

**Introduction:**

The conceptualization and protective mechanisms of resilience may differ across age groups, leaving the underlying processes of resilience against learning burnout among pupils largely unexplored. According to Kumpfer’ s resilience framework, resilience in pupils depends on the successful adaptation of resiliency characteristics (e.g., academic self-efficacy) to their environment (e.g., school adjustment).

**Methods:**

To examine the mediating roles of both factors, 413 pupils (49.6% male; M = 10.81, SD = 0.72) from Shenzhen, China, participated. SEM with bootstrapping was used to test the mediation model.

**Results:**

(1) consistent with findings in older students, school adjustment mediated the relationship between resilience and learning burnout; (2) In contrast to older students, academic self-efficacy did not function as an independent mediator, as it did not significantly predict learning burnout. Instead, it exerted its protective effect indirectly through a sequential pathway involving school adjustment.

**Conclusion:**

These findings suggest that pupils’ resilience may rely more on the school, with academic self-efficacy buffering learning burnout only when it supports adaptive functioning in school. Early interventions that strengthen internal resources and promote constructive school adjustment may thus help mitigate learning burnout in this age group.

## 1 Introduction

Learning burnout, a widespread issue characterized by emotional and physical exhaustion, reduced accomplishment, and avoidance of school tasks due to excessive academic pressure, is associated with various psychological and behavioral problems among students, parents, and teachers, and further undermines overall educational quality (Schaufeli et al., 2002; Madigan and Curran, 2021; Madigan and Kim, 2021; Wu et al., 2022). Given the various problems related to learning burnout, understanding the protective factors and its underlying mechanism is essential. However, existing research has predominantly focused on older students, such as those in secondary schools and universities (e.g., Madigan et al., 2024; Cai et al., 2025), with limited attention to primary school pupils. There are several reasons to focus on learning burnout among Chinese pupils, and the insights gained may offer implications for primary education in other contexts. First, learning burnout is relatively common among pupils in China. Under the traditional Chinese cultural expectation of “making sons succeed and daughters excel,” pupils are often burdened with academic demands, particularly through extracurricular tutoring (Tao and Xu, 2022). A latent profile study reported that two-thirds of Chinese pupils can be classified into the burnout group (Yang et al., 2023). Second, as pupils are still in an early stage of development, their coping strategies are less mature than those of older students (Sotardi, 2016), which makes them more vulnerable to learning burnout and its detrimental effects. Existing evidence indicates that learning burnout in pupils is associated with poorer sleep quality, reduced learning motivation, and dissatisfaction with teaching (Qin et al., 2022; Usán et al., 2022; Yang et al., 2023). Third, this developmental period also presents a crucial window for prevention. Pupils are at a critical stage of development (Bizzi et al., 2022), and previous trajectory research suggest that early intervention can reduce the risk of adverse developmental pathways of learning burnout, especially in primary school (Vansoeterstede et al., 2023; Parviainen et al., 2021). Therefore, understanding the underlying mechanisms of protective factors against learning burnout, such as resilience, is particularly crucial among pupils in China and other cultural contexts.

Resilience, as the capacity for adaptation in the face of adversity, has been widely shown to protect students against learning burnout (Vansoeterstede et al., 2023). However, an important research gap remains: resilience may manifest in fundamentally different ways in pupils compared to older students. Consequently, the underlying mechanisms linking resilience to burnout in pupils may differ, and resiliency processes observed in older students may not directly apply to this age group. Resilience is a broad construct, and its meaning vary across developmental stages (Lerner et al., 2012). A systematic review suggests that resilience shifts from adaptive functioning across individual and immediate environments in school-aged children (6–12 years) to broader socio-ecological resources that promote well-being during adolescence (13–19 years) (Yoon et al., 2021). Building on this, Kumpfer’s (2002) resilience framework emphasizes that resilience develops through dynamic interactions between individual resiliency characteristics and risk / protective environmental factors, which together shape adaptive functioning. When learning burnout is considered as a form of chronic academic stress, academic self-efficacy can be viewed as a key individual characteristic that enables pupils to cope with academic stress, while school adjustment, though measured at the individual level, reflects pupils’ adaptation to the school environment and peer relationships, thus serving as an indicator of the reconstruction of school environment (Wentzel, 2003; Travis et al., 2020; Moss, 2021). Together, these factors could capture core resilience mechanisms among pupils and highlight critical developmental differences in resilience. According to developmental perspectives, although pupils may not yet possess the autonomy of older students to actively mobilize socio-ecological resources (Beyers et al., 2025), those with stronger confidence in their abilities tend to develop greater internal motivation, enabling them to better socialize and adapt within their most immediate developmental context (i.e., the school), ultimately helping them cope more effectively with current developmental pressures during Erikson’s industry vs. inferiority stage (Erikson, 1963; Reeve et al., 2020). In other words, pupils who demonstrate higher academic self-efficacy and better school adjustment can be understood as more resilient individuals against learning burnout.

Therefore, the present study addresses this gap by examining the associations of resilience with learning burnout and the potential mediating roles of academic self-efficacy and school adjustment among Chinese pupils. Focusing on this younger age group provides a developmental perspective on how resilience relates to learning burnout and offers empirical evidence to inform the design of resilience-based interventions tailored to primary school contexts.

### 1.1 Academic self-efficacy as a mediator

It’s reasonable to believe that resilience positively relates to academic self-efficacy. Academic self-efficacy refers to an individual’s confidence in successfully completing academic tasks (Bandura, 1997). Resilience has been shown to have a robust positive relationship with general self-efficacy in both intervention and correlational meta-analyses, suggesting that academic self-efficacy, as a domain-specific construct, may demonstrate a similar association among pupils (Lee et al., 2013; Gallagher et al., 2020; Wu et al., 2023). According to Kumpfer’s (2002) resilience framework, resilience is shaped by individual resiliency characteristics (i.e., cognitive, emotional, and behavioral strengths) that interact with environmental factors to promote adaptive outcomes. Academic self-efficacy can be understood as a key cognitive component through which resilience operates in the academic domain (Benight and Cieslak, 2011). Specifically, resilient students possess psychological resources such as positive cognition, emotional control, and goal setting, which enable them to accumulate repeated mastery experiences in learning and maintain a stable mental and physical state, thereby strengthening their confidence in academic tasks (Parsons et al., 2016; Gong et al., 2021). At the same time, supportive family and peer relationships as their resilient micro-environment, provide opportunities for vicarious experiences and verbal persuasion, further enriching the sources of efficacy beliefs among students (Kleppang et al., 2023). Through these pathways, pupils can effectively draw on Bandura’s (1997) proposed sources of self-efficacy, ultimately fostering higher levels of academic self-efficacy. Focusing on academic self-efficacy as a specific domain, a longitudinal study indicate that general resilience factors play an important role in helping primary and secondary school students maintain academic self-efficacy under adversity (Repo et al., 2025); meanwhile, experimental evidence shows that college students exposed to adverse stimuli demonstrate a positive association between resilience and academic self-efficacy (Cassidy, 2015).

Moreover, academic self-efficacy may negatively relate to learning burnout. According to Bandura’s (2001) social cognitive theory, human behavior is shaped by the dynamic interaction among cognitive, behavioral, and environmental factors. As a core cognitive factor of the social cognitive theory, academic self-efficacy influences students’ learning behaviors, including task selection, effort investment, persistence, and the use of coping strategies (Schunk and DiBenedetto, 2022). Students with higher academic self-efficacy are more likely to engage actively in learning, cope effectively with academic challenges, and regulate their effort and persistence to manage academic demands, thereby reducing the risk of learning burnout (Yang and Tu, 2025). Previous quasi-experimental and longitudinal studies among older students provide evidence suggesting this causal interpretation that increases in academic self-efficacy (or self-efficacy more broadly) may help reduce learning burnout (Bresó et al., 2011; Huang et al., 2023).

Therefore, despite most evidence coming from older students, academic self-efficacy may similarly mediate the link between resilience and learning burnout among pupils.

### 1.2 School adjustment as a mediator

Resilience may positively relate to school adjustment. Although school adjustment lacks a universally accepted definition (Wentzel, 2003), following the research tradition that conceptualizes it as a multidimensional construct, it refers in this study to students’ adaptive functioning within the school environment, encompassing psychological and behavioral adjustment, peer and teacher-student relationships, and academic adjustment. Resilience in pupils, conceptualized as adaptive functioning across immediate microsystems (Yoon et al., 2021), is likely to contribute to better school adjustment, as school serves as a primary setting for socialization. Kumpfer’s (2002) resilience framework emphasizes that resilience emerges from effectively utilizing protective factors and managing risks in environments, making school adjustment a key indicator of how resilience manifests. Specifically, resilient students’ psychological resources (e.g., positive cognition, emotion regulation, and goal setting) enable them to engage effectively in learning tasks, regulate behavior, and maintain positive attitudes and emotions toward school, while supportive family and peer relationships provide guidance, encouragement, and role models, facilitating better interactions with teachers and classmates (Caleon and King, 2020; Williams and Anthony, 2015). Although causal evidence is limited, cross-sectional studies show a positive association between resilience and school adjustment among older students (Azpiazu et al., 2024), and a longitudinal evidence reported that school adjustment predicts subsequent psychological outcomes following adversity, indicating that resilience may influence later mental health in part through its effect on school adjustment (Leonard and Gudiño, 2021).

Additionally, because school is the immediate environment where learning burnout emerges, school adjustment is likely to negatively predict learning burnout. According to ecological systems theory, school represents one of the most important microsystems for the development of children and adolescents (Bronfenbrenner, 2000). In school, students with better school adjustment can build stable and harmonious interactions with teachers, classmates, and academic tasks, thereby creating a supportive environment that reduces stress and strengthens their sense of competence and belonging (Choi et al., 2023). In this way, higher levels of school adjustment function as a protective factor against the development of learning burnout. A longitudinal study based on high school students supports the positive effect of school adjustment on learning burnout (Tang et al., 2024).

Therefore, although direct evidence from pupils is limited, it is reasonable to assume that school adjustment may serve a mediating role between resilience and learning burnout.

### 1.3 Academic self-efficacy and school adjustment: a chain of mediation

Lastly, we propose a chain mediation through academic self-efficacy and school adjustment. According to Kumpfer’s (2002) resilience framework, resilient individuals can mobilize protective resources (e.g., academic self-efficacy) within their environments (e.g., school) while reinterpreting and reconstructing risk factors, thereby gaining adaptive advantages through person–environment interactions. Bandura’s (2001) social cognitive theory further explains this process: through triadic reciprocal determinism, academic self-efficacy functions as a core cognitive factor that shapes how students perceive and respond to their school environment. By fostering a positive cognitive framework, academic self-efficacy enables students to view school as controllable and to approach academic pressures and interpersonal challenges as manageable tasks rather than threats, thereby promoting better adjustment across psychological, relational, and academic domains (Jungert and Rosander, 2010). Although direct evidence for this causal explanation is lacking, with only cross-sectional studies demonstrating a positive association between academic self-efficacy and school adjustment (Campos et al., 2022), several longitudinal studies have shown that academic self-efficacy positively predicts students’ psychological well-being in school as well as their academic and interpersonal adaptation (Thomas et al., 2022; Kristensen et al., 2023; Affuso et al., 2025). These findings provide indirect support for the proposed chain mediation in which academic self-efficacy facilitates school adjustment.

### 1.4 The present study

**Figure 1:**
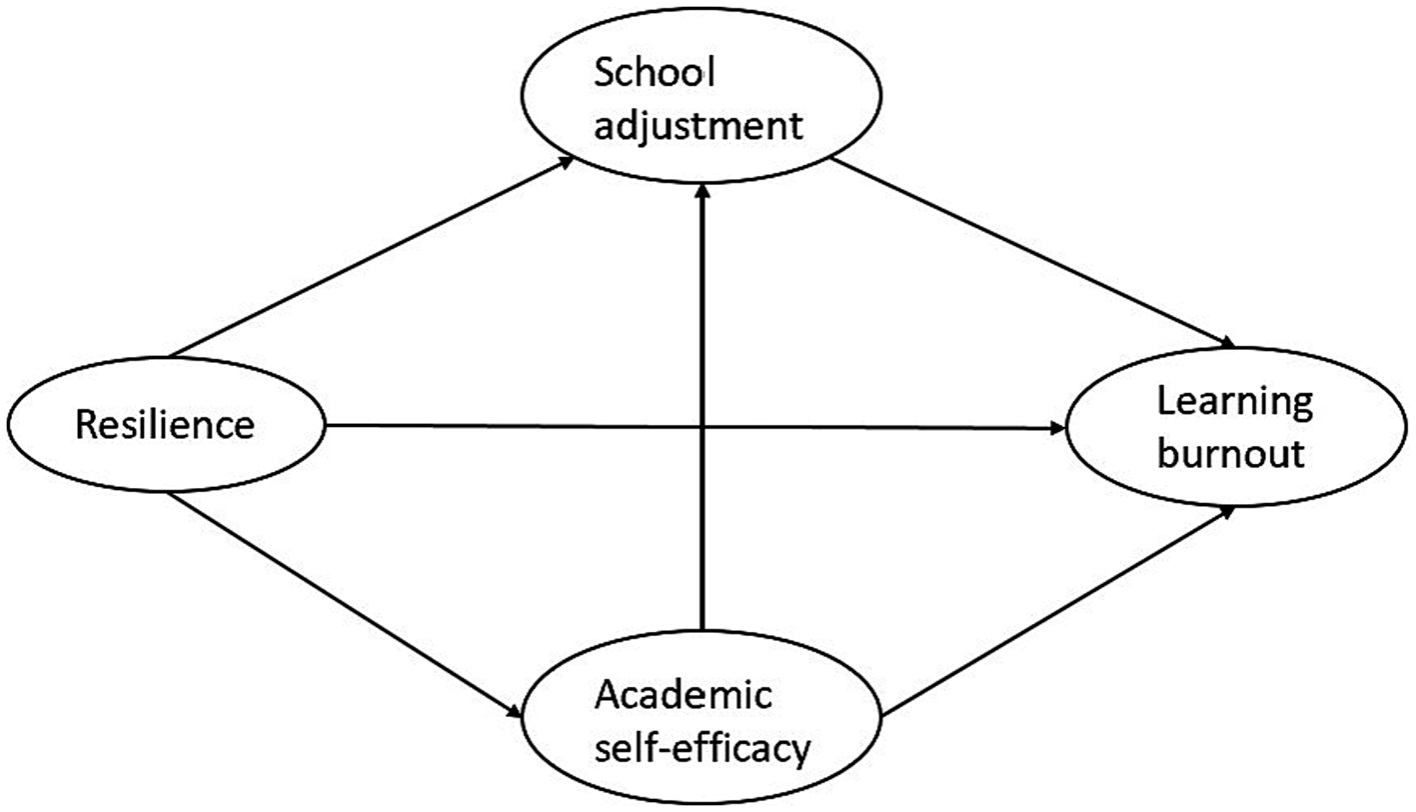
The conceptual model.

In the existing literature, little attention has been paid to the underlying mechanisms linking resilience to learning burnout among pupils. To address this research gap, and building on the theoretical frameworks discussed above, we hypothesize that academic self-efficacy and school adjustment capture key processes through which resilience operates in the school context, and ultimately protects pupils from learning burnout. Accordingly, the present study proposed a multiple mediation model for exploration in Figure 1. It is noteworthy that recent studies have highlighted debates regarding the factor structure of burnout (Bianchi et al., 2019; Wang et al., 2024). When burnout is conceptualized as a process of stress development, individuals typically experience emotional exhaustion first under excessive job demands, whereas depersonalization and reduced personal accomplishment can be viewed as coping reactions and subsequent outcomes of exhaustion (Leiter, 2018). From this perspective, exhaustion is often regarded as the core component of burnout and may further evolve into depressive symptoms (Schonfeld and Bianchi, 2016). Although some empirical evidence supports the lagged effects of exhaustion on depersonalization and reduced personal accomplishment (e.g., Diestel and Schmidt, 2010), the conceptual debate on the dimensionality of burnout remains unresolved (Schaufeli, 2021). In addition, academic self-efficacy may conceptually overlap with the reduced personal accomplishment dimension of burnout. As clarifying the factor structure of burnout is not the primary aim of this study, we examined both the three-factor model and an alternative model using exhaustion alone as the dependent variable to ensure the robustness of our findings. The specific hypotheses are as follows:

*Hypothesis 1*: Resilience negatively relates to learning burnout.

*Hypothesis 2*: Academic self-efficacy mediates the relationship between resilience and learning burnout.

*Hypothesis 3*: School adjustment mediates the relationship between resilience and learning burnout.

*Hypothesis 4*: Academic self-efficacy and school adjustment sequentially mediate the relationship between resilience and learning burnout.

## 2 Method

### 2.1 Participants

Ethical approval was granted by the ethical committee of authors’ institution. Written informed consent was obtained from both pupils and their legal guardians in line with ethical standards for research with minors. Of the 454 pupils invited via convenience sampling from a elementary school in Shenzhen, China. The selected primary school is located in Shenzhen, one of China’s most economically developed and socially competitive cities, in an area with a high proportion of migrant families. This context provides a representative environment for studying learning burnout among pupils in China, combining intense academic competition with the challenges faced by children from migrant backgrounds (23.9% of participants come from other cities). However, our sample may still not be generalizable to all Chinese pupils outside this context. Trained graduate students explained the study’s purpose, procedures, and confidentiality using age-appropriate language during class. Surveys were conducted in classrooms of 40–50 pupils, supervised by a trained postgraduate and the regular teacher to assist as needed. The questionnaire was pilot-tested with a similar age group to ensure clarity. Pupils were informed they could skip questions or withdraw freely. Completion took about 30 min. Forty-one responses were excluded due to extreme response patterns and a large number of missing items. Four hundred thirteen fifth- and sixth-grade pupils (*M* = 10.81, SD = 0.72, ages 10–13, 49.6% male, 50.4% female) were remained as participants.

### 2.2 Measurements

#### 2.2.1 Resilience

The Resilience Scale for Chinese Adolescents (RSCA, Hu and Gan, 2008) aligns with the conceptualization of resilience in pupils, that is, adaptive functioning across individual psychological, family, and peer domains. It captures both individual psychological resources and support from family and peers. The scale consists of 27 items rated on a 5-point Likert scale (1 = never, 5 = always) and includes five dimensions: goal focus (e.g., “I set goals and enjoy working toward them,” *α* = 0.70), emotional control (e.g., “I can quickly regulate my emotions,” *α* = 0.79), positive cognition (e.g., “I believe everything has a positive side,” *α* = 0.70), family support (e.g., “My family values my opinions,” *α* = 0.73), and interpersonal assistance (e.g., “I have a friend to share my troubles with,” *α* = 0.71). Total scores range from 52 to 153, with higher scores indicating greater resilience. Moreover, this scale has been widely used in China and has demonstrated good internal consistency in previous studies involving Chinese school-aged populations (Ran et al., 2022; Peng et al., 2024).

#### 2.2.2 Learning burnout

Learning burnout was measured using the Adolescent Student Burnout Inventory (ASBI; Wu et al., 2010), a 16-item scale adapted from the Maslach Burnout Inventory (Maslach et al., 1997) for Chinese adolescents in the academic context, comprising three dimensions and rated on a 5-point Likert scale (1 = not at all true, 5 = very true). The dimensions are physical and emotional exhaustion (e.g., “Recently, I have felt very empty inside,” *α* = 0.78), avoidance of school tasks (e.g., “I am failing my schoolwork and want to quit,” *α* = 0.70), and reduced personal accomplishment (e.g., “I cannot feel a sense of achievement in learning,” *α* = 0.81), with the latter reverse scored. In this study, total burnout scores ranged from 20 to 70, with higher scores indicating greater risk of learning burnout. The scale showed good internal consistency in previous studies on Chinese school-aged populations (Zhou et al., 2024; Zhang et al., 2025).

#### 2.2.3 Academic self-efficacy

Based on Gibson and Dembo’s (1984) original scale, we used the revised Chinese version of the Academic Self-efficacy Scale developed by Zhou and Dong (1994). The scale includes 12 items forming six indicators, with each indicator consisting of one positively worded item and one reverse-scored item. These six indicators are divided evenly into two dimensions: learning behavior self-efficacy (three indicators, e.g., “Studying science courses like math is difficult for me,” and “I believe I can excel in science courses like math,” *α* = 0.65, McDonald’s *ω* = 0.72) and learning ability self-efficacy (three indicators, e.g., “I can handle the learning problems I come across,” and “I find studying really hard,” *α* = 0.68, McDonald’s *ω* = 0.74). Each indicator score is calculated as the mean of its positive and reverse-scored items. Items are rated on a 6-point Likert scale (1 = not at all true, 6 = very true), and total scores ranged from 19 to 98.5, with higher scores indicating greater academic self-efficacy. The scale was applied in Chinese pupils before and showed good internal consistency (Tan et al., 2017). Although the Cronbach’s *α* of the two subscales were slightly below the conventional 0.70 threshold, the appropriate cutoff for reliability depends on research purposes and stage (Cho and Kim, 2015). To the best of our knowledge, in the absence of well-validated measures of academic self-efficacy for pupils, an α around the more lenient threshold of 0.60 can still be considered acceptable (Taber, 2018). Moreover, the McDonald’s ω coefficients exceeded 0.70, suggesting acceptable internal consistency. This apparent discrepancy between *α* and *ω* can be attributed to the different assumptions underlying the two indices: Cronbach’s *α* assumes tau-equivalence (equal factor loadings), which is often violated in psychological constructs with heterogeneous items, whereas ω takes into account the actual factor loadings obtained from the model and thus provides a more accurate reliability estimate when items contribute unequally to the latent construct (Kalkbrenner, 2024). Nevertheless, despite the overall stability of the factor structure, we interpreted this construct with appropriate caution.

#### 2.2.4 School adjustment

The Middle School Student’s Adaptability Scale (Cui, 2008) assesses school adjustment with 27 items across five dimensions using a 5-point Likert scale (1 = not at all true, 5 = very true). The dimensions include peer relationships (e.g., “My classmates do not like me,” *α* = 0.83), teacher-student relationships (e.g., “I avoid meeting my teachers,” *α* = 0.84), academic adjustment (e.g., “I often get sidetracked when studying,” *α* = 0.71), regular adjustment (e.g., “I am often punished for being undisciplined,” *α* = 0.73), and emotions and attitudes toward school (e.g., “I wish I did not have to go to school,” *α* = 0.84). Some items were reverse scored to ensure validity. Total scores ranged from 39 to 185, with higher scores indicating better school adjustment. This scale showed good internal consistency in previous studies of Chinese school-aged populations (Bai et al., 2022; Yu et al., 2025).

#### 2.2.5 Demographic factors

We collected participants’ demographic information, including age, gender, and parents’ educational levels, through a self-designed questionnaire and treated these variables as controls in the analyses.

#### 2.2.6 Common method biases

To examine potential common method bias, we applied the unmeasured latent method variable (ULMV) approach by contrasting a bifactor model that included a common method factor with a single-factor model without it (Williams and McGonagle, 2016). The analysis revealed that the bifactor model exhibited poorer model fit relative to the single-factor model (ΔRMSEA = 0.08, ΔCFI = −0.045, ΔTLI = −0.049, ΔSRMR = 0.01). These findings suggest that common method bias is unlikely to pose a serious issue in the present research.

#### 2.2.7 Analysis progress

We first conducted correlation analysis, reliability analysis using SPSS 22.0. Subsequently, structural equation modeling (SEM) was performed with Mplus 7.4. To test the significance of the mediation effects, a bias-corrected bootstrap procedure with 5,000 resamples was applied.

## 3 Results

### 3.1 Description and correlation

Table 1 presents the means and standard deviations for resilience, learning burnout, school adjustment, and academic self-efficacy. All core variables were significantly correlated with each other.

**Table 1:** Descriptive statistics and correlation matrix.

Variables	*M*	SD	1	2	3	4
1. Resilience	3.43	0.56	—			
2. Learning burnout	2.29	0.59	−0.69***	—		
3. School adjustment	3.91	0.59	0.45***	−0.51***	—	
4. Academic self-efficacy	4.03	0.76	0.54***	−0.56***	0.50***	—

*N* = 413.

*p < 0.05, **p < 0.01, ***p < 0.001.

M, mean; SD, standard deviations.

### 3.2 Testing the structural equation model

**Figure 2:**
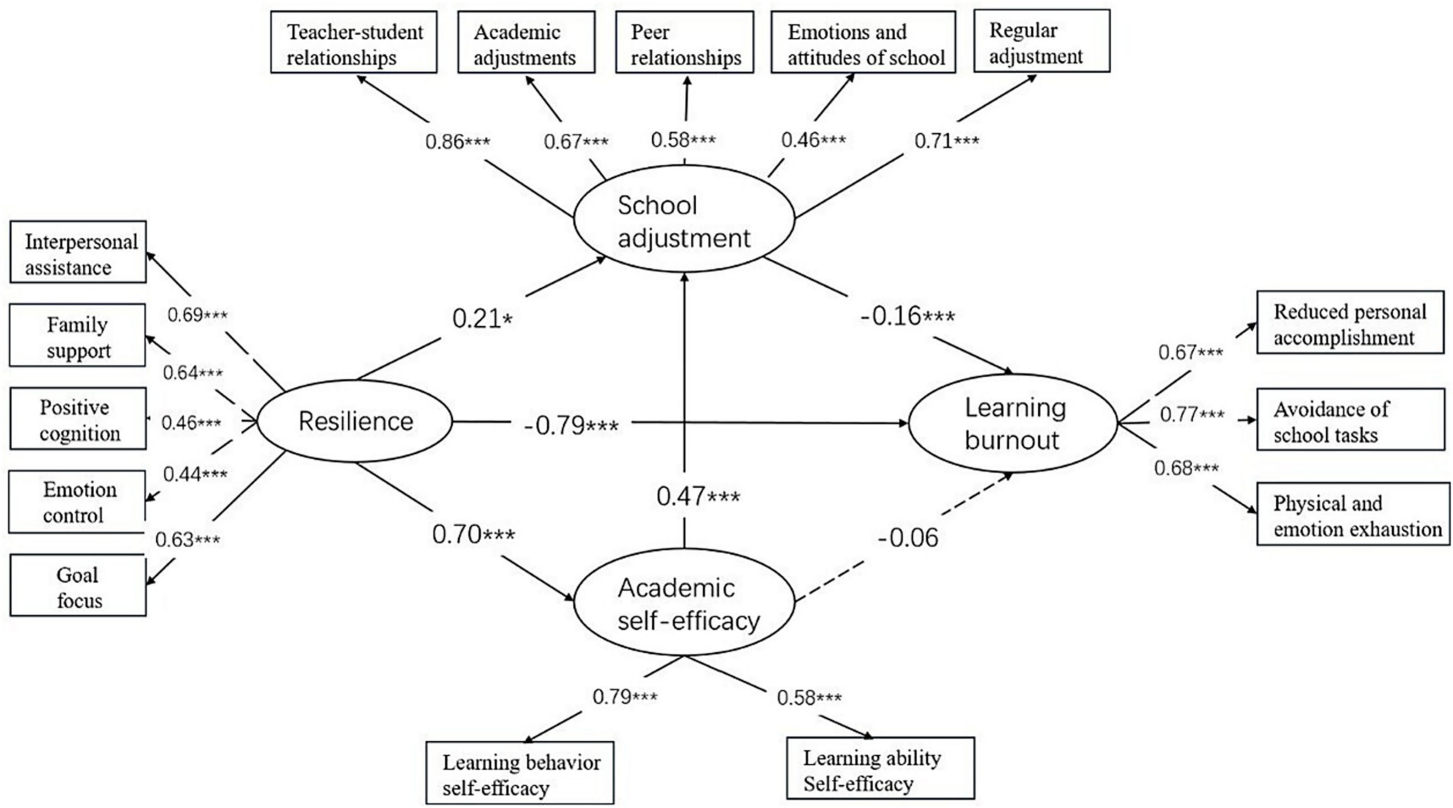
Examination of the structural equational model. *N* = 413, *p < 0.05, **p < 0.01, ***p < 0.001.

After controlling for demographic variables, the model shown in Figure 2 demonstrated a good fit to the data (*χ*^2^ = 232.45, df = 78, *χ*^2^/df = 2.98, *p* < 0.001; CFI = 0.926; TLI = 0.901; RMSEA = 0.069; SRMR = 0.044). No significant associations were found between the control variables and the key variables. As shown in Figure 2, resilience negatively predicted learning burnout (*β* = −0.79, *p* < 0.001) and positively predicted academic self-efficacy (*β* = 0.70, *p* < 0.001) and school adjustment (*β* = 0.21, *p* < 0.05). Academic self-efficacy further predicted school adjustment (*β* = 0.47, *p* < 0.001), while school adjustment also predicted lower burnout (*β* = −0.16, *p* < 0.001). However, no significant association between academic self-efficacy and learning burnout (*β* = −0.06, *p* > 0.05). Furthermore, as shown in Table 2, school adjustment mediated the relationship between resilience and learning burnout (*β* = −0.03, 95% CI = [−0.21, −0.01]), and academic self-efficacy and school adjustment sequentially mediated this relationship (*β* = −0.05, 95% CI = [−0.12, −0.01]). Nevertheless, the indirect effect of academic self-efficacy was insignificant (*β* = −0.05, 95% CI = [−0.09, 0.08]).

**Table 2:** Testing the pathways of the multiple mediation model.

Path	*β*	95% Confidence interval	
		Lower	Upper
a. Total effect model			
Resilience → Learning burnout	−0.93^a^	−0.98	−0.83
b. Multiple mediation model			
Direct effects			
Resilience → Learning burnout	−0.79^a^	−0.94	−0.63
Resilience → School adjustment	0.21^a^	0.004	0.40
Resilience → Academic self-efficacy	0.70^a^	0.63	0.78
School adjustment → Learning burnout	−0.16^a^	−0.29	−0.02
Academic self-efficacy → Learning burnout	−0.06	−0.30	0.11
Academic self-efficacy → School adjustment	0.47^a^	0.63	0.78
Indirect effects			
Resilience → School adjustment → Learning burnout	−0.03^a^	−0.21	−0.00
Resilience → Academic self-efficacy → Learning burnout	−0.05	−0.09	0.08
Resilience → Academic self-efficacy → School adjustment → Learning burnout	−0.05^a^	−0.12	−0.01

^a^95% confidence interval does not overlap with zero.

**Figure 3:**
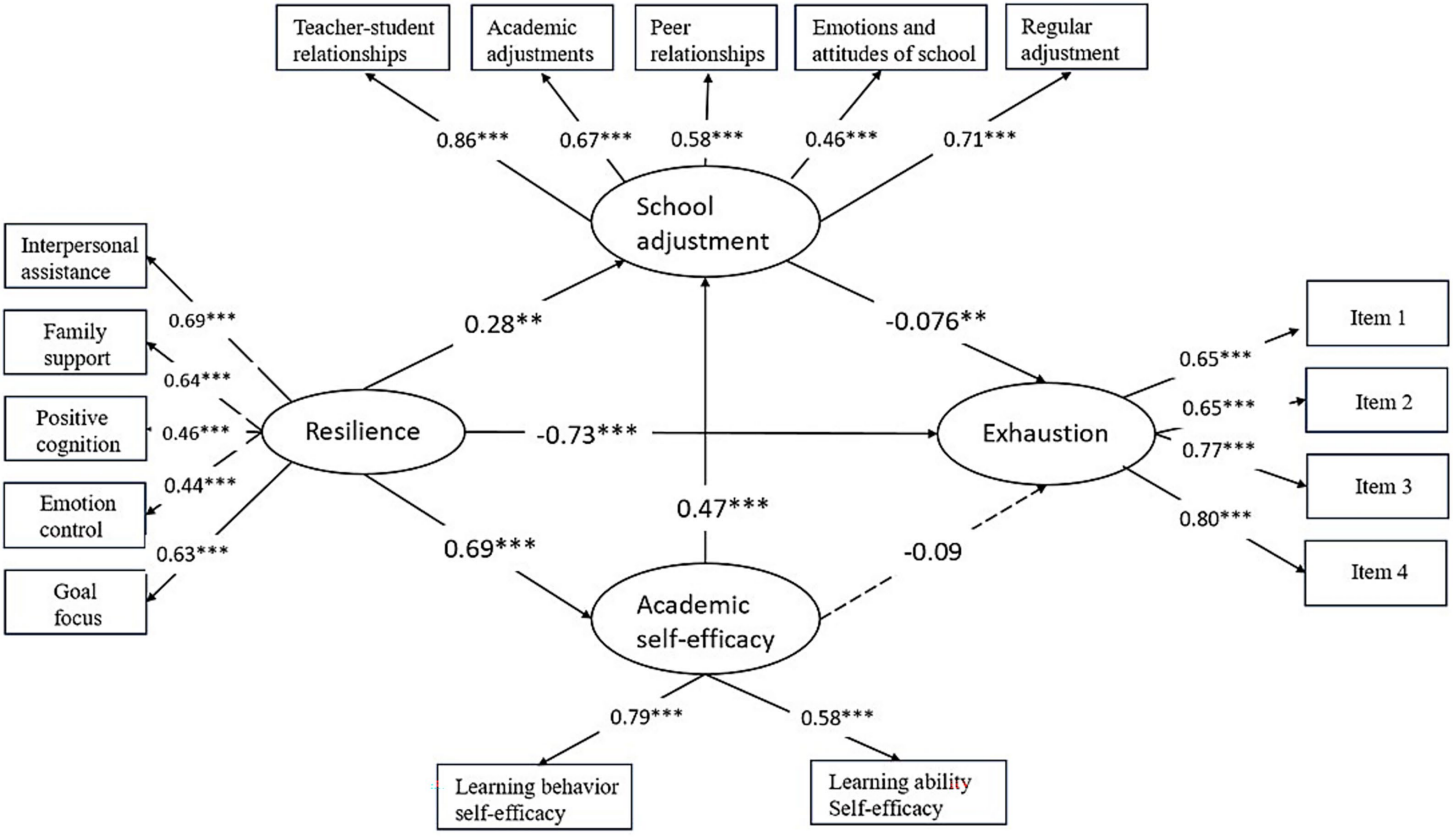
Examination of the alternative model. *N* = 413, *p < 0.05, **p < 0.01, ***p < 0.001.

Building on recent findings that exhaustion as the core component of burnout (Bianchi et al., 2019), we further examined whether our model remained robust when exhaustion was treated as the outcome variable. To this end, we tested an alternative model using the four exhaustion items as observe variables. The alternative model showed a slightly poorer yet acceptable fit (*χ*^2^ = 319.397, df = 98, *χ*^2^/df = 3.26, *p* < 0.001; CFI = 0.903; TLI = 0.891; RMSEA = 0.075; SRMR = 0.06). Although minor differences emerged in the effect size and significance of some path coefficients, the overall pattern of relationships among the constructs remained consistent. These results suggest that our original model is robust, and therefore, we retained it for subsequent analyses (Figures 2, 3).

## 4 Discussion

Although resilience has been shown to protect older students from learning burnout, little is known about the mechanisms underlying this association in primary school pupils, whose resilience may operate differently due to their different developmental stage. To address this gap, the present study found that: (1) consistent with findings in older students, school adjustment mediated the relationship between resilience and learning burnout; (2) in contrast to older students, academic self-efficacy did not function as an independent mediator, as it did not significantly predict learning burnout. Instead, it exerted its protective effect indirectly through a sequential pathway with school adjustment. Moreover, the alternative model tested in response to debates on the burnout factor structure indicated the robustness of our findings. These results suggest that among pupils, resilience may reduce learning burnout primarily through enhanced adaptive functioning within the school environment, with internal resiliency factor as academic self-efficacy likely contributing indirectly via external school adjustment. In the following discussion, we focus on burnout as an overall construct rather than its internal structure, avoiding speculative interpretations of its dimensions.

### 4.1 Mediating role of school adjustment

Aligning with previous cross-sectional and longitudinal evidence from older students (Leonard and Gudiño, 2021; Azpiazu et al., 2024; Tang et al., 2024), the present study found that school adjustment mediated the relationship between resilience and learning burnout among pupils. Our findings support the use of Kumpfer’s (2002) resilience framework to understand how resilience functions in primary school pupils, a specific developmental stage, in relation to learning burnout. This framework emphasizes that the protective role of resilience depends on dynamic interactions between resilient individuals and their environment. Specifically, resilience in pupils reflects adaptive functioning across individual, family, and peer domains (Yoon et al., 2021). Given that school is one of the microsystems for their development (Bronfenbrenner, 2000), when stressors arise from academic demands, highly resilient pupils are able to leverage their cognitive and emotional resources, supported by family and peers, to actively address risks in the school environment and recognize protective factors. Through these constructive person–environment interactions, pupils can achieve adaptive functioning across multiple domains within the school context.

Additionally, trajectory studies have revealed unfavorable developmental patterns of learning burnout, with an intensifying trend particularly during the middle school years (Vansoeterstede et al., 2023; Parviainen et al., 2021). Scholars have argued that as students progress through the educational system, schools often struggle to adequately meet their evolving psychological needs, which may lead to growing feelings of alienation and helplessness in learning (Eccles and Roeser, 2015). In this context, given that the protective role of resilience against learning burnout largely depends on the extent to which students adapt to the school environment, schools bear a particularly important responsibility. Implementing school-based resilience interventions at the primary school stage may not only help mitigate students’ current academic stress but also serve as a long-term preventive measure, reducing the likelihood that learning burnout will intensify during middle school.

### 4.2 Mediating role of academic self-efficacy

Although it was theoretically expected that academic self-efficacy would mediate the relationship between resilience and learning burnout, this indirect pathway was not supported in our sample of pupils. While the positive association between resilience and academic self-efficacy is consistent with longitudinal and experimental evidence from older students (Cassidy, 2015; Repo et al., 2025), the finding that academic self-efficacy did not significantly predict learning burnout diverges from prior evidence in older student populations (Bresó et al., 2011; Huang et al., 2023). The role of self-efficacy in shaping behavior is well-established in social cognitive theory. Therefore, our findings suggest 2 possible explanations, both of which remain valid when considering the alternative model.

First, measurement factors may obscure the relationship between academic self-efficacy and learning burnout in this age group. Our alternative model, which excluded potential conceptual overlap between the factor structure of burnout and academic self-efficacy, allows us to focus primarily on the measurement of academic self-efficacy. Bandura (2012) emphasizes that self-efficacy is context-specific, varying across domains and even within facets of a domain, which means measurement issues can contribute to inconsistent findings. Although we measured domain-specific academic self-efficacy, the scale was not originally designed for pupils, and nearly half of the items were reverse-scored. This may introduce method effects similar to those observed in the Rosenberg self-esteem scale, which tend to be amplified in children (Marsh et al., 2010). Empirical evidence indicates that children’s ability to respond consistently to negatively worded items changes with age; items designed to measure the same construct in both positive and negative wording are often uncorrelated in younger children but become increasingly correlated in older children (Marsh, 1986). In other words, differences in language comprehension and cognitive development may have introduced measurement artifacts. Our findings further indicated that, despite the acceptable McDonald’s *ω*, the Cronbach’s *α* of this instrument fell below the conventional threshold, implying that internal consistency was somewhat limited and that the effect of academic self-efficacy on learning burnout might have been underestimated. Therefore, the non-significant direct association between academic self-efficacy and learning burnout may partly reflect methodological limitations.

Second, although academic self-efficacy was significantly correlated with learning burnout in the bivariate analyses, this direct path became non-significant in the SEM after including school adjustment. This suggests that the protective role of academic self-efficacy likely operates indirectly through school adjustment, consistent with the chain mediation effect observed in our results.

### 4.3 Chain mediating effects of academic self-efficacy and school adjustment

In line with previous longitudinal evidence suggesting indirect associations (Thomas et al., 2022; Kristensen et al., 2023; Affuso et al., 2025), although our cross-sectional design cannot capture the dynamic process of chain mediation, the theoretical framework provides a plausible explanation for the observed pattern. Kumpfer’s (2002) resilience framework emphasizes that resilience does not merely stem from internal traits but emerges through individuals’ active mobilization and integration of protective and risk factors within their environments, thereby achieving adaptive outcomes. From this perspective, academic self-efficacy can be understood as a core cognitive resource, yet its protective function against learning burnout is unlikely to operate directly and instead relies on interaction with the school environment. Bandura’s (2001) social cognitive theory further elaborates this mechanism through the principle of triadic reciprocal determinism: human functioning results from the interplay of personal factors, behaviors, and environmental influences. The environment is not a fixed force but a set of potentials that must be selected and activated by the individual to shape developmental trajectories (Bandura, 2012). Specifically, academic self-efficacy, as part of students’ internal resources, provides them with a positive cognitive framework that enables them to reinterpret academic pressures and interpersonal challenges as manageable and controllable tasks, thereby fostering school adjustment. The analysis above resonates with evidence from a meta-analysis showing that while self-efficacy and academic achievement are reciprocally related in adults, such a direct reciprocal link was not found in children (Talsma et al., 2018). Although that study focused on achievement rather than burnout, the implication is informative: if self-efficacy in childhood is insufficient to directly enhance positive learning outcomes such as achievement, it is even less likely to directly buffer against negative learning outcomes such as burnout. Instead, its protective effect must be channeled through adaptive processes like school adjustment, thereby lending indirect support to the proposed chain mediation. In other words, school adjustment functions as a crucial bridge in pupils’ resiliency process, linking the cognitive resources of academic self-efficacy to the reduction of learning burnout.

In sum, consistent with the conceptualization of resilience in pupils, the protective process against learning burnout does not rest solely on academic self-efficacy as an individual characteristic. From a developmental perspective, it is crucial for pupils at this stage to develop confidence in their abilities (Erikson, 1963). More resilient pupils may exhibit a level of autonomy akin to adolescents, proactively adapting to their environment and mobilizing resources to meet challenges (see relative evidence in Zhao et al., 2024 and Reeve et al., 2020). At the same time, given that their abstract thinking is still developing (Piaget, 1952), pupils are more likely to link their learning abilities to concrete aspects of their school experience (such as peers, teachers, classroom activities, and tasks), rather than to abstract feelings of burnout. Crucially, even when considering that exhaustion is often regarded as the core component of learning burnout (Schonfeld and Bianchi, 2016), this interpretation remains valid. Academic self-efficacy likely operates primarily through motivational and coping mechanisms (e.g., engagement) (Zhen et al., 2020), rather than directly alleviating exhaustion itself. Because exhaustion is a highly abstract affective-cognitive composite state (Schaufeli, 2021), pupils may have limited capacity for recognizing or articulating it, relying instead on tangible indicators of adjustment in their daily school experience. Empirical evidence indicates that depressive symptoms, conceptually situated on the same continuum as burnout, exert a lagged effect on self-efficacy rather than the reverse direction in early adolescence (Tak et al., 2017). Thus, the protective effect of academic self-efficacy is more likely to operate through concrete school adjustment processes rather than directly alleviating abstract learning burnout. However, given the cross-sectional design, these pathways should be interpreted as correlational rather than causal, and future longitudinal work is needed to clarify their temporal order.

### 4.4 Practical implications

Given evidence that learning burnout may increase in later school stages, early attention to pupils’ adaptation within the school environment could be particularly valuable (Vansoeterstede et al., 2023). As researchers have noted, the relevance of protective factors may vary across developmental stages, and resilience-focused interventions should be tailored to age-appropriate factors to align with targeted psychological outcomes and students’ developmental needs, thereby potentially enhancing preventive effects (Dray et al., 2017). In light of the promising prospects of school-based resilience interventions, these findings offer preliminary insights for educational practice.

First, school adjustment appears to play a central role in the relationship between resilience and learning burnout among primary school pupils. This suggests that supportive learning environments (characterized by positive teacher–student interactions, opportunities for peer collaboration, and practices that foster students’ sense of competence) may help pupils adaptively cope with academic challenges (see relative discussions in Reeve, 2013). For example, allowing students to ask questions, choose their learning partners, and select from various tasks or levels of challenge has been shown to foster an autonomy-supportive school climate, which in turn promotes students’ overall development (Hastie et al., 2013).

Second, although academic self-efficacy is positively associated with resilience, its protective effect against learning burnout seems to operate indirectly, likely through school adjustment. Therefore, resilience interventions may be most effective when they combine strategies to strengthen self-efficacy with activities that promote adaptive functioning in the school environment (see relative discussions in Maddux, 2013). Potential practical directions include scaffolded goal-setting around academic tasks, which allows students to experience mastery in manageable steps and strengthens their confidence in academic abilities; collaborative learning tasks and role-play activities, which help students familiarize themselves with school norms and build positive interpersonal relationships; and practicing emotion regulation in classroom contexts, supported by positive feedback, to enhance students’ attitudes and engagement with school.

### 4.5 Limitations

There are several limitations to our study. First, due to its cross-sectional design, causal relationships cannot be established. For example, it is equally plausible that poor school adjustment could be an antecedent, rather than a consequence, of declining academic self-efficacy, particularly when the school environment fails to meet pupils’ emerging needs for autonomy development. Future longitudinal studies are essential to establish temporal precedents. Second, all data were based on pupils’ self-reports, excluding input from key stakeholders such as parents and teachers, which limits ecological validity. Third, the sample was geographically restricted to pupils in Shenzhen, China, thereby constraining the generalizability of the findings. Future cross-cultural comparisons or studies in less developed regions of China could provide further valuable insights.

More importantly, as Bandura (2012) emphasized, self-efficacy is a highly context-specific construct, which makes its measurement particularly challenging. The academic self-efficacy scale used in this study was not originally developed or fully validated for young children, potentially introducing method effects, although these were not detected in our tests for method bias. Therefore, the insignificant direct effect of academic self-efficacy on burnout may partly reflect measurement limitations rather than true developmental differences. Future research using age-appropriate, validated self-efficacy measures is needed to clarify this distinction. The development and validation of a specific academic self-efficacy scale for elementary school students is an important next step for this field.

## 5 Conclusion

Few studies have examined the resilience process of pupils facing learning burnout, whose resilience mechanisms may differ fundamentally from those of older students, while they are both more vulnerable and at a critical stage for prevention. According to Kumpfer’s framework, we examine the mediating roles of individual characteristics (e.g., academic self-efficacy) and of adaptation to the school environment (e.g., school adjustment). This study found that:

1. Consistent with findings in older students, school adjustment mediated the relationship between resilience and learning burnout;
2. In contrast to older students, academic self-efficacy did not function as an independent mediator, as it did not significantly predict learning burnout. Instead, it exerted its protective effect indirectly through a sequential pathway involving school adjustment.

These findings suggest that pupils’ resilience may rely more on the school environment, with academic self-efficacy only buffering learning burnout when it enhances adaptive functioning in school. Early interventions that strengthen internal resources may depend on promoting constructive school adjustment in order to help mitigate learning burnout in this age group.

## Data Availability

The datasets presented in this article are not readily available because the data are not publicly available due to privacy and ethical restrictions. Requests to access the datasets should be directed to Qiufeng Gao, gqf_psy@szu.edu.cn.
