# Correction: Exploring resilience mechanism in learning burnout among pupils: school adjustment and academic self-efficacy

**DOI:** 10.3389/fpsyg.2025.1748106

**Published:** 2025-11-26

**Authors:** Changcheng Jiang, Qiufeng Gao

**Affiliations:** School of Government, Shenzhen University, Shenzhen, China

**Keywords:** learning burnout, resilience, school adjustment, academic self-efficacy, pupils

Changcheng Jiang was erroneously assigned as corresponding author. The correct corresponding author is Qiufeng Gao.

The original version of this article has been updated.

